# Phase-Specific Biomechanical Reorganization After Robotic Rehabilitation in Patients with Stroke: A Sensor-Derived Waveform Analysis

**DOI:** 10.3390/life16060956

**Published:** 2026-06-05

**Authors:** Hande Argunsah, Hülya Şirzai, Yigit Can Gökhan, Güneş Yavuzer, Köksal Holoğlu

**Affiliations:** 1 Department of Biomedical Engineering, Faculty of Engineering and Natural Sciences, Acibadem Mehmet Ali Aydinlar University, 34755 Istanbul, Turkey; yigit.gokhan@live.acibadem.edu.tr; 2 Department of Biomedical Engineering, Graduate School of Natural and Applied Sciences, Acibadem Mehmet Ali Aydinlar University, 34755 Istanbul, Turkey; 3 Romatem Move Physical Therapy and Rehabilitation Hospital, 34692 Istanbul, Turkey; hulyasirzai@romatem.com (H.Ş.); gunesyavuzer@romatem.com (G.Y.); koksal@romatem.com (K.H.)

**Keywords:** robotic rehabilitation, stroke, trunk stability, waveform analysis, clustering, sensor-based assessment

## Abstract

Stroke-related gait impairments are frequently associated with deficits in trunk control, movement coordination, and dynamic stability. Although robotic-assisted gait rehabilitation has shown promising clinical benefits, phase-specific biomechanical adaptations following rehabilitation remain incompletely understood. This study investigated phase-specific biomechanical adaptations following robotic-assisted gait rehabilitation in individuals with stroke using sensor-derived waveform analysis. Rehabilitation was performed three times per week over approximately 5–6 weeks using treadmill-based robotic gait training under dynamic body-weight support conditions. Pre- and post-intervention kinematic data were collected using a sensor-based motion analysis system. Joint kinematics, trunk motion, and center of gravity (COG) displacement were analyzed across the normalized gait cycle using waveform-based effect size analysis, statistical parametric mapping, principal component analysis, and k-means clustering to explore inter-individual adaptation patterns. Thirteen post-stroke hemiplegia patients (10 males; age = 63.9 ± 13.8 years), including six subacute and seven chronic stroke survivors, completed 16 rehabilitation sessions. The most prominent improvements were observed in trunk lateral flexion, particularly during loading response (d = 0.47, *p* < 0.01), indicating enhanced frontal plane trunk stability. Trunk flexion–extension showed reduced compensatory motion, whereas hip and knee adaptations were smaller and phase-dependent. COG displacement decreased across the gait cycle, reflecting improved dynamic stability. Step length increased significantly on both hemiplegic (Δ = +5.73 cm, *p* = 0.024) and intact sides (Δ = +8.83 cm, *p* = 0.007), while cadence and load symmetry remained unchanged. Clustering analysis revealed heterogeneous adaptation profiles rather than distinct responder groups. Chronic participants demonstrated greater variability within the Principal Component Analysis space compared to subacute participants, suggesting more variable and individualized biomechanical reorganization patterns rather than clearly separable recovery categories. Overall, robotic rehabilitation induced inter-individual biomechanical adaptations, predominantly involving proximal trunk control and stabilization strategies.

## 1. Introduction

Stroke remains one of the leading causes of long-term disability worldwide and is frequently associated with persistent impairments in gait and postural control. These impairments extend beyond reductions in walking speed and endurance, encompassing deficits in intersegmental coordination, trunk stability, and symmetrical weight transfer. Impaired trunk control has been identified as a critical determinant of gait dysfunction and balance deficits in neurological populations, influencing dynamic stability and functional mobility during walking [1,2].

Robotic-assisted gait training has emerged as a promising intervention to enhance motor recovery by providing high-intensity, repetitive, and task-specific movement practice. Modern rehabilitation robots can deliver adaptive training through human–robot interaction mechanisms, thereby promoting motor learning and neuroplasticity [3,4]. In addition, advances in control strategies, such as impedance-based and adaptive control approaches, have further improved the responsiveness and personalization of robotic rehabilitation systems [5]. A growing body of evidence, including systematic reviews and meta-analyses, supports the effectiveness of robot-assisted gait training in improving gait performance, balance, and kinematic outcomes in individuals with stroke and other neurological disorders [6,7,8].

Despite these advancements, the evaluation of rehabilitation outcomes remains largely dependent on discrete or averaged biomechanical and clinical parameters, such as gait speed, cadence, or balance scores. While these measures provide valuable clinical insights, they fail to capture the dynamic and phase-dependent nature of human gait. Human locomotion is inherently cyclical and requires precise coordination across different phases of the gait cycle, each characterized by distinct biomechanical demands. Consequently, motor recovery following rehabilitation is unlikely to occur uniformly across the entire gait cycle but rather manifests as phase-specific adaptations reflecting selective improvements in motor control [9,10]. Analyzing gait using continuous waveform-based approaches may therefore provide more detailed insight into localized biomechanical adaptations and compensatory movement strategies that are not detectable using conventional discrete metrics [10,11,12].

Recent developments in wearable sensor technologies and motion analysis systems have enabled continuous monitoring of movement patterns, offering new opportunities to assess gait in a more comprehensive manner [13,14,15,16]. Sensor-based approaches have been increasingly used to quantify gait and balance performance in neurological populations and have demonstrated strong potential for capturing subtle changes in motor behavior that are not detectable using conventional metrics [9,10,11].

Previous studies have shown that wearable sensor systems can effectively quantify gait asymmetry, temporal variability, dynamic stability, and altered movement coordination following stroke [11,12,13,16]. Sensor-derived analyses have also provided valuable insight into compensatory gait strategies and phase-dependent motor alterations in post-stroke locomotion [11,12,13]. However, most previous studies have primarily focused on discrete spatiotemporal parameters or cycle-averaged kinematic outcomes, potentially overlooking localized biomechanical adaptations occurring within specific phases of the gait cycle. Therefore, an important knowledge gap remains regarding how robotic rehabilitation influences continuous gait waveforms and whether these adaptations occur uniformly or in a phase-specific manner throughout the gait cycle. Specifically, we aimed to (1) quantify the magnitude of pre–post changes across the gait cycle using waveform-based metrics, (2) evaluate alterations in movement patterns through waveform similarity analysis, and (3) explore inter-individual adaptation patterns using exploratory unsupervised clustering techniques. We hypothesized that biomechanical changes following robotic rehabilitation would not be uniformly distributed across the gait cycle and that waveform-based analysis would reveal both magnitude and pattern-level adaptations indicative of motor reorganization.

## 2. Materials and Methods

### 2.1. Participants

Individuals with post-stroke hemiplegia undergoing robotic rehabilitation at a specialized neurorehabilitation center were recruited for this study. Based on time since stroke, patients were categorized into three phases: acute (0–7 days), subacute (7 days–6 months), and chronic (>6 months). Stroke patients were selected according to predefined clinical and functional criteria to ensure that all participants were suitable candidates for robotic gait rehabilitation. Inclusion criteria required: (i) a confirmed diagnosis of stroke; (ii) the ability to ambulate with minimal assistance or under partial body-weight support; (iii) sufficient cognitive capacity to understand and follow verbal instructions; and (iv) medical clearance from a rehabilitation physician for participation in robotic gait training. Exclusion criteria included: (i) severe musculoskeletal deformities (e.g., fixed contractures) that would interfere with treadmill-based gait training; (ii) uncontrolled cardiopulmonary or metabolic disease; (iii) pronounced spasticity or pain limiting safe participation; and (iv) comorbidities such as vestibular disorders or acute orthopedic injuries that could confound gait assessment. All patients were ambulatory with minimal assistance and were able to complete the 16-session robotic gait training protocol. All participants provided written informed consent prior to participation. The study protocol was approved by the institutional ethics committee (approval no: 2024-KAEK-37) and conducted in accordance with the principles of the Declaration of Helsinki.

### 2.2. Study Protocol and Data Analysis

Participants underwent a standardized robotic-assisted gait training program consisting of 16 sessions. Each session involved task-specific gait training using the Tecnobody Smart Gravity Walker (Tecnobody, Bergamo, Italy). The system integrates an optical markerless motion analysis system that records multi-joint kinematics using depth sensors and infrared-based tracking. Kinematic data were sampled at 100 Hz, with an angular resolution of 0.1° and a spatial resolution of approximately 1 mm.

Sessions were conducted three times per week, with each lasting approximately 60 min. Accordingly, the total rehabilitation duration was approximately 5–6 weeks depending on scheduling and patient availability. Each rehabilitation session consisted of task-specific treadmill-based robotic gait training performed under dynamic body-weight support conditions. Training began with a warm-up phase under stable body-weight support, followed by a progressive gait-training period in which treadmill speed, unloading percentage, and gait difficulty were adjusted according to each participant’s functional capacity and clinical progression. Initial treadmill speeds ranged between 0.4 and 0.8 m/s and were gradually increased in increments of 0.1–0.2 m/s based on gait stability, stepping rhythm, safe foot placement, and patient tolerance. Body-weight support levels were progressively reduced throughout the intervention as motor control and balance improved. All sessions were supervised by an experienced physiotherapist who continuously monitored patient safety, fatigue, cardiovascular response, and gait performance. Rest periods were provided when necessary to avoid excessive fatigue and ensure safe participation throughout the rehabilitation protocol.

All sessions were supervised by an experienced physiotherapist, who monitored patient safety and adjusted parameters based on fatigue, cardiovascular response, and motor performance. Emergency stop systems and dynamic harness support were used continuously to ensure safe participation. Kinematic data were collected before and after the intervention using the system’s integrated motion analysis module. The recorded parameters included bilateral knee flexion–extension, bilateral hip flexion–extension, trunk flexion–extension, trunk lateral flexion, and COG displacement (cm). Each waveform was time-normalized to 100% of the gait cycle and averaged across several consecutive strides to obtain representative patterns. This normalization procedure ensured that variations in walking speed and stride duration did not influence the temporal alignment of the data. As a result, waveform comparisons between pre- and post-rehabilitation conditions could be performed on a point-by-point basis.

To investigate whether biomechanical changes were localized within specific portions of the gait cycle, the normalized waveform was divided into five functional phases initial contact (0–20%), loading response (20–40%), mid-stance (40–60%), terminal stance (60–80%) and swing phase (80–100%). For each phase, mean values were computed for both pre- and post-rehabilitation conditions. Phase-specific differences were then calculated to identify localized adaptations in gait mechanics. In addition to continuous waveform analysis, discrete features were extracted from each parameter to facilitate statistical comparisons and support secondary analyses. These features provided complementary information and enabled integration with conventional statistical approaches commonly used in clinical biomechanics.

### 2.3. Statistical Analysis

All statistical analyses were performed using IBM SPSS Statistics 30 and Python 3.12 and SciPy 1.16.3. Data normality was assessed using the Shapiro–Wilk test. For normally distributed variables, paired *t*-tests were used to compare pre- and post-rehabilitation values. For non-normally distributed data, the Wilcoxon signed-rank test was applied. Effect sizes were calculated using Cohen’s d to quantify the magnitude of changes. Statistical significance was set at *p* < 0.05. Effect size maps were visualized as continuous profiles to identify the regions of strong adaptation and phase-specific recovery patterns.

To investigate inter-individual variability in biomechanical adaptations following robotic rehabilitation, a patient-specific clustering analysis was performed. For each participant, mean changes (post–pre) across the normalized gait cycle were calculated for five biomechanical parameters: trunk flexion–extension, trunk lateral flexion, hip flexion–extension, knee flexion–extension, and center of gravity (COG) displacement. These features represent overall directional changes in kinematic behavior following the intervention. Prior to analysis, all features were standardized using z-score normalization to ensure comparability across variables with different units and scales. Principal component analysis (PCA) was then applied to reduce data dimensionality and to identify the dominant patterns of variability within the dataset. The first two principal components (PC1 and PC2) were retained for visualization and interpretation.

Subsequently, k-means clustering was performed on the standardized feature matrix to identify subgroups of patients with similar adaptation profiles. A two-cluster solution (k = 2) was selected based on silhouette analysis and interpretability considerations given the sample size. The quality of clustering was evaluated using the silhouette coefficient. To facilitate interpretation of the principal components, a loadings analysis was conducted, indicating the contribution of each biomechanical variable to PC1 and PC2. All analyses were performed using Python-based statistical tools.

## 3. Results

The study cohort consisted of thirteen post-stroke hemiplegia patients (3 females, 10 males; mean age = 63.9 ± 13.8 years), including six subacute and seven chronic stroke participants (Table 1). The results are presented to characterize biomechanical adaptations following robotic-assisted gait rehabilitation using waveform-based, discrete, and similarity analyses. Time-normalized waveforms were examined to identify global and phase-specific changes, followed by effect size and statistical analyses to localize adaptations across the gait cycle.

**Figure 1:**
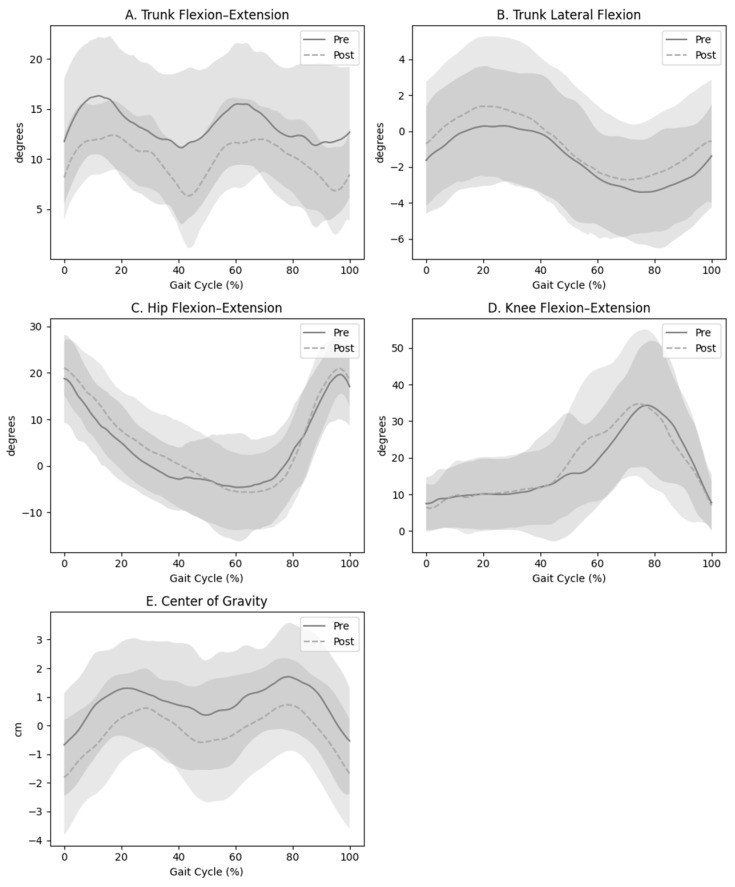
Mean time-normalized waveforms (0–100% gait cycle) for pre-rehabilitation and post-rehabilitation across all parameters. Solid lines represent the mean waveform profiles, whereas the surrounding light and dark gray shaded regions indicate the corresponding ±1 standard deviation (1σ) variability envelopes for each condition. The lighter shaded area corresponds to the pre-rehabilitation variability envelope, whereas the darker shaded area represents the post-rehabilitation variability envelope.

Time-normalized waveform analysis revealed phase-dependent biomechanical adaptations following robotic rehabilitation, with consistent but parameter-specific changes observed between pre- and post-intervention conditions (Figure 1). In trunk flexion–extension, post-rehabilitation waveforms demonstrated a general reduction in amplitude and variability across the gait cycle, suggesting improved movement consistency and a decrease in compensatory trunk strategies. Similarly, trunk lateral flexion showed a shift toward reduced lateral deviation, particularly during mid-stance and late stance phases, indicating improved frontal plane trunk control and enhanced postural alignment. In hip flexion–extension, post-rehabilitation waveforms exhibited a more refined and smoother pattern rather than a marked increase in overall excursion. Subtle changes were observed during the stance-to-swing transition, suggesting improved intersegmental coordination and more efficient limb advancement. Knee flexion–extension displayed relatively minor waveform alterations, with only modest differences during the swing phase, supporting the notion that distal joint kinematics remain less responsive to the intervention compared to proximal segments. COG displacement demonstrated a tendency toward reduced variability and a more compact trajectory across the gait cycle following rehabilitation. This pattern reflects improved dynamic stability and more controlled whole-body motion, aligning with the observed improvements in trunk control.

**Figure 2:**
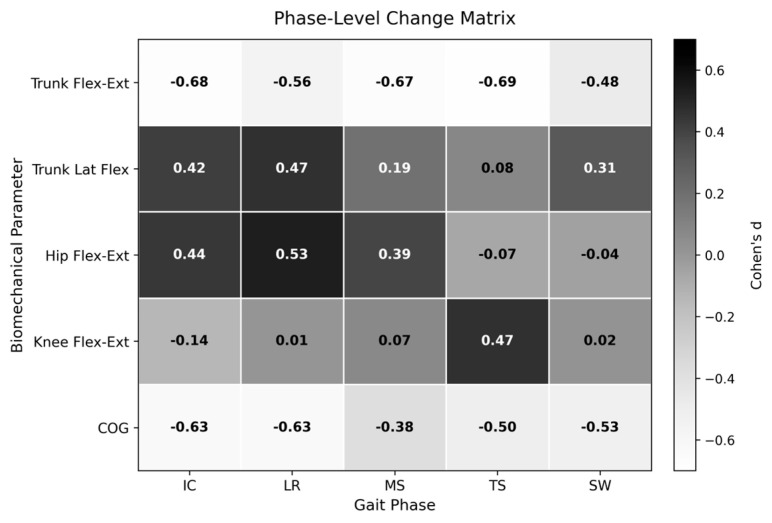
Phase-level change matrix illustrating the magnitude of pre–post biomechanical adaptations across the gait cycle. Effect sizes (Cohen’s d) are presented for five functional gait phases: initial contact (IC), loading response (LR), mid-stance (MS), terminal stance (TS), and swing (SW). Positive values indicate an increase, whereas negative values indicate a decrease following rehabilitation.

Figure 2 provides a phase-resolved summary of pre–post biomechanical changes across the gait cycle, highlighting where rehabilitation-induced adaptations are concentrated. The most prominent and consistent effects are observed in trunk flexion–extension, which demonstrates moderate-to-large negative effect sizes across all phases (d ≈ −0.48 to −0.69). This indicates a systematic reduction in trunk motion amplitude throughout the gait cycle, suggesting improved trunk stability and a reduction in compensatory sagittal plane movements following rehabilitation. Trunk lateral flexion exhibits moderate positive effect sizes, particularly during early stance phases (initial contact and loading response; d ≈ 0.42–0.47). These changes reflect a shift toward improved frontal plane alignment and reduced pathological lateral deviation, indicating enhanced postural control during weight acceptance and early single-limb support.

For hip flexion–extension, moderate positive effects are primarily observed during initial contact through mid-stance (d ≈ 0.39–0.53), suggesting improved proximal joint contribution during weight acceptance and support. However, near-zero to slightly negative effects in terminal stance and swing indicate phase-dependent reorganization rather than a uniform increase in range of motion. Knee flexion–extension shows relatively small and phase-specific changes, with the most notable increase during terminal stance (d = 0.47), while other phases demonstrate minimal effects. This pattern indicates that distal joint adaptations remain limited and less responsive to the intervention compared to proximal segments. Finally, COG displacement demonstrates consistent moderate negative effect sizes across all phases (d ≈ −0.38 to −0.63), reflecting a reduction in movement amplitude and variability. This suggests improved dynamic stability and more controlled whole-body motion throughout the gait cycle, without a strong phase-specific peak but rather a global stabilization effect.

Pre–post comparisons of spatiotemporal and kinematic parameters are presented in Table 2. Overall, most discrete kinematic variables demonstrated small and non-significant changes following the intervention (*p* > 0.05). Hip flexion–extension remained largely unchanged (Δ = +0.35°, *p* = 0.901, d = 0.04), while knee flexion–extension showed a modest, non-significant increase (Δ = +2.49°, *p* = 0.398, d = 0.24). Similarly, trunk flexion–extension exhibited a small increase (Δ = +0.99°, *p* = 0.276, d = 0.32), whereas trunk lateral flexion remained essentially unchanged (Δ = −0.05°, *p* = 0.943, d = −0.02). COG displacement also demonstrated negligible differences between conditions (Δ = +0.01 cm, *p* = 0.945, d = 0.02). In terms of spatiotemporal parameters, cadence showed no statistically significant change following rehabilitation (*p* = 0.982), with only minimal overall differences between conditions. Contact times for both limbs and load symmetry exhibited non-significant alterations with small-to-moderate effect sizes (*p* > 0.05), indicating limited changes in temporal gait characteristics.

In contrast, step length demonstrated the most pronounced improvements. Step length on the hemiparetic side increased significantly (Δ = +5.73 cm, *p* = 0.024, d = 0.75), accompanied by a substantial relative change (+65.8%). Similarly, step length on the intact side showed a significant increase (Δ = +8.83 cm, *p* = 0.007), reflecting improved stride symmetry and gait efficiency, with a moderate effect size (d = 0.58).

**Figure 3:**
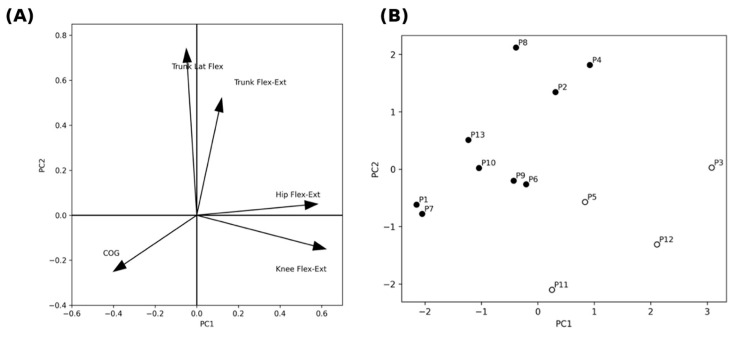
Patient-specific biomechanical adaptation patterns following robotic rehabilitation. (**A**) PCA loadings plot illustrating the contribution of each biomechanical variable to the principal components. Arrows represent the direction and magnitude of variable loadings. PC1 is primarily associated with lower limb kinematics (hip and knee flexion–extension), reflecting distal movement adaptations, whereas PC2 is dominated by trunk-related variables (trunk flexion–extension and lateral flexion), representing proximal control mechanisms. (**B**) Cluster distribution of participants (P1–P13) based on PCA and k-means clustering applied to patient-specific changes in biomechanical parameters. Each point represents an individual participant. Filled circles indicate Cluster 1, whereas open circles indicate Cluster 2.

Patient-specific clustering analysis was performed to explore inter-individual variability in biomechanical adaptations following robotic rehabilitation (Figure 3). The two-cluster solution yielded a modest silhouette score (0.22–0.25), indicating limited separation between groups and suggesting overlapping adaptation patterns across patients. Rather than forming clearly distinct subgroups, patients were distributed along a continuum within the principal component space. Visual inspection of the PCA distribution revealed variability in the direction and magnitude of biomechanical changes across individuals, without sharp boundaries between clusters. Additionally, chronic stroke participants appeared to exhibit a broader spatial distribution within the PCA feature space compared to subacute participants, suggesting greater inter-individual variability in biomechanical adaptation patterns following rehabilitation. This observation is consistent with previous studies reporting increased movement heterogeneity, compensatory motor behavior, and individualized gait strategies in chronic stroke populations compared to earlier recovery stages [11,12,16]. Chronic stroke survivors often demonstrate more variable biomechanical adaptations due to long-term motor compensation and prolonged neuromuscular reorganization processes [11,12]. This pattern indicates that biomechanical adaptations following robotic rehabilitation may be relatively more homogeneous in subacute stroke and more variable in chronic stroke. However, these observations should be interpreted cautiously given the limited sample size and the exploratory nature of the clustering analysis.

Principal component analysis showed that the first two components explained approximately 69% of the total variance (PC1: 42.2%, PC2: 26.8%). Loadings analysis indicated that PC1 was primarily associated with lower limb kinematics, particularly hip and knee flexion–extension, whereas PC2 was dominated by trunk-related variables, including trunk flexion–extension and lateral flexion.

At the individual level, patients demonstrated heterogeneous adaptation profiles across biomechanical parameters. Some individuals exhibited more pronounced changes in trunk-related variables, while others showed relatively greater changes in lower limb kinematics. Similarly, reductions in COG displacement were more evident in certain patients, whereas others demonstrated minimal changes in this parameter. Although k-means clustering partitioned the data into two groups, these clusters largely reflected differences in the relative contribution of biomechanical parameters rather than clearly separable response categories. This pattern indicates that rehabilitation-induced adaptations vary continuously across individuals, rather than conforming to discrete subgroups.

## 4. Discussion

This study investigated phase-specific biomechanical adaptations following robotic-assisted gait rehabilitation in individuals with stroke using a sensor-derived approach. The main findings indicate that rehabilitation-induced changes are not uniformly distributed across the gait cycle but instead exhibit clear phase-dependent and segment-specific patterns. In particular, the most consistent adaptations were observed in trunk kinematics, especially in the frontal plane, while distal joints demonstrated more limited and variable changes. In addition, clustering analysis revealed inter-individual variability in adaptation profiles rather than clearly separable subgroups, highlighting the presence of heterogeneous response patterns across patients.

One of the most notable findings of this study is the consistent modification in trunk lateral flexion across gait phases, with the strongest effect observed during loading response. This phase corresponds to weight acceptance and is critical for maintaining mediolateral stability. The observed reduction in pathological lateral deviation suggests improved postural alignment and enhanced proximal control. These findings are consistent with previous studies emphasizing the central role of trunk stability in gait recovery after stroke, where improved trunk control has been associated with better balance and functional mobility [17,18]. From a neuromechanical perspective, these results indicate that robotic rehabilitation may preferentially facilitate proximal stabilization mechanisms, contributing to more efficient control of body mass during stance [19,20,21,22].

In contrast, trunk flexion–extension demonstrated a consistent reduction across the gait cycle, reflected by moderate negative effect sizes. This reduction represents a stabilization strategy characterized by decreased excessive trunk motion. In post-stroke gait, exaggerated trunk movements are often used as compensatory mechanisms to maintain balance and forward progression. Therefore, the observed decrease in sagittal trunk excursion may reflect a transition toward more controlled and energy-efficient movement patterns.

Joint-level adaptations in the lower limbs were more modest and phase-dependent. Hip flexion–extension exhibited moderate changes primarily during loading response and mid-stance, suggesting enhanced contribution to weight acceptance and forward propulsion. However, the presence of phase-specific variability indicates that these adaptations reflect a redistribution of movement rather than a uniform increase across the gait cycle. Similarly, knee flexion–extension showed only small-to-moderate improvements, particularly during swing and terminal stance. This limited responsiveness of distal joints compared to proximal segments aligns with previous evidence suggesting that recovery of coordinated distal motor control is more challenging and often delayed following stroke [23,24].

The analysis of COG displacement further supports the interpretation of improved dynamic stability. The observed reduction in COG amplitude suggests improved dynamic stability and more efficient control of whole-body motion during gait [16]. Importantly, these changes were distributed across the gait cycle rather than confined to specific phases, indicating a global stabilization effect rather than localized adaptations.

Interestingly, discrete and spatiotemporal parameters did not show widespread significant changes, except for step length. Both hemiplegic and intact sides demonstrated substantial increases in step length, with large effect sizes and statistical significance, particularly on the intact side. In contrast, cadence, contact time, and load symmetry remained largely unchanged. This suggests that improvements in gait performance were achieved primarily through spatial reorganization rather than temporal modulation. In other words, patients primarily demonstrated spatial adaptations in gait pattern rather than substantial temporal reorganization. This dissociation between spatial and temporal parameters highlights the importance of evaluating gait beyond conventional summary metrics.

A key contribution of this study lies in the application of waveform-based analysis, which enabled the identification of phase-specific adaptations that would not be detectable using traditional discrete measures alone. By preserving the temporal structure of gait, waveform-based approaches enable identification of localized biomechanical adaptations that may remain undetected when gait is summarized using cycle-averaged discrete metrics [11]. Importantly, waveform-based phase-specific adaptations do not necessarily correspond directly to changes in discrete whole-cycle parameters, as localized reductions within specific gait phases may coexist with small increases in the overall mean waveform value. Therefore, the apparent differences between waveform-derived and discrete summary metrics should not be interpreted as contradictory findings, but rather as complementary representations of different aspects of gait adaptation. The findings demonstrate that rehabilitation effects are concentrated in specific phases and primarily involve proximal control mechanisms.

The present findings are generally consistent with previous studies reporting that robotic-assisted gait rehabilitation can improve gait stability, trunk control, and movement coordination in individuals with stroke [3,19,20]. Prior sensor-based studies have demonstrated that wearable motion analysis systems can detect gait asymmetry, compensatory movement strategies, and dynamic stability alterations following stroke [11,12,13,16]. However, most previous investigations primarily focused on discrete spatiotemporal metrics or averaged kinematic parameters, which may not fully capture localized adaptations occurring within specific phases of the gait cycle. In contrast, the present study applied waveform-based phase-specific analysis combined with exploratory multivariate biomechanical characterization to investigate how robotic rehabilitation modifies continuous gait patterns across the normalized gait cycle. Therefore, this study extends the current literature by demonstrating that rehabilitation-related biomechanical adaptations after stroke are not uniformly distributed throughout the gait cycle but are instead concentrated within specific gait phases, particularly involving proximal trunk control and stabilization mechanisms. Additionally, the exploratory PCA-based findings suggest that chronic stroke participants may exhibit more variable and individualized adaptation patterns, highlighting the potential value of temporally resolved biomechanical analysis for characterizing inter-individual recovery variability following robotic rehabilitation.

Furthermore, the exploratory clustering analysis revealed parameter-specific heterogeneity in patient responses rather than clearly defined responder subgroups. While group-level analyses suggested modest average changes, the PCA-based representation showed that patients were distributed along a continuum within the feature space, reflecting variability in both the magnitude and direction of biomechanical adaptations; therefore individual patients exhibited different combinations of trunk, lower limb, and COG-related changes. In this context, the first principal component (PC1) was primarily associated with lower limb kinematics, particularly hip and knee flexion–extension, whereas the second component (PC2) was dominated by trunk-related variables. This indicates that patient-specific adaptations can be interpreted along two main dimensions: distal kinematic changes and proximal control mechanisms. Accordingly, some patients demonstrated more pronounced adaptations in trunk-related parameters, suggesting a stabilization-driven response, whereas others exhibited relatively greater changes in hip and knee kinematics, reflecting a locomotor-oriented adaptation pattern. Importantly, these differences should be interpreted as variations along a continuous spectrum rather than discrete recovery categories. Additionally, the relatively low silhouette scores and limited sample size indicate substantial overlap between participants, further supporting the interpretation that these patterns reflect continuous inter-individual variability rather than robustly separable biomechanical subgroups. Therefore, the clustering analysis should be considered exploratory and hypothesis-generating rather than confirmatory.

Importantly, integration of clinical data into the PCA space and cluster composition analysis provided additional context for interpreting these patterns. Given the absence of clearly separable clusters and the modest clustering quality metrics, these observations should be interpreted cautiously, and trends related to stroke chronicity and clinical severity should be considered exploratory. Although these trends were not statistically significant, they suggest that stroke chronicity and clinical severity may influence the degree of biomechanical reorganization following rehabilitation.

When the PCA distribution was examined according to stroke phase, subacute participants tended to demonstrate a relatively more compact distribution pattern, whereas chronic participants showed greater dispersion across the feature space. This observation suggests that patients in the subacute stage may respond to robotic rehabilitation with more homogeneous biomechanical adaptations, potentially reflecting relatively more homogeneous recovery-related adaptation patterns during the earlier stages of post-stroke neuroplasticity. In contrast, chronic stroke survivors appeared to exhibit more individualized and variable adaptation profiles, likely reflecting compensatory movement strategies and long-term motor reorganization processes.

Crucially, this pattern should not be interpreted as greater overall recovery in chronic patients, but rather as a difference in adaptation across stroke phases. In early stages (acute and subacute), recovery is largely driven by spontaneous neurobiological processes and restitution of impaired motor function, resulting in more global but relatively homogeneous improvements. In contrast, chronic patients, whose spontaneous recovery has plateaued, may exhibit greater biomechanical reorganization through compensatory and strategy-level adaptations. In this context, greater dispersion in the PCA space may reflect increased variability in movement strategies rather than superior functional recovery.

This study has several limitations that should be considered when interpreting the findings. First, the sample size was relatively small, limiting the generalizability of the results and reducing the statistical robustness of subgroup-level interpretations. In particular, the distribution of participants across stroke phases may have influenced the observed variability patterns; therefore, findings related to subacute and chronic stroke should be interpreted cautiously. Additionally, although both ischemic and hemorrhagic stroke survivors were included, subgroup analyses based on stroke type were not performed due to the limited sample size. The present study primarily focused on stroke phase-related biomechanical adaptations rather than etiological subgroup comparisons.

Second, the absence of a non-robotic rehabilitation comparison group limits the ability to distinguish rehabilitation-specific biomechanical adaptations from general recovery-related changes. Third, although waveform-based analysis provides detailed phase-specific information regarding gait biomechanics, the present study focused primarily on kinematic parameters and did not include electromyographic, kinetic, or neurophysiological measurements that could further explain the underlying mechanisms of motor reorganization.

Furthermore, the exploratory clustering analysis should be interpreted cautiously due to the limited sample size and modest silhouette scores, which indicate overlapping adaptation patterns rather than clearly separable patient subgroups. Therefore, the Principal Component Analysis- and clustering-based findings should be considered exploratory and hypothesis-generating rather than confirmatory.

Finally, long-term follow-up assessments were not performed; therefore, the persistence and clinical relevance of the observed biomechanical adaptations remain unclear. Future studies involving larger cohorts, multimodal biomechanical assessments, control-group comparisons, and longitudinal follow-up designs are needed to better characterize individualized recovery trajectories following robotic rehabilitation after stroke.

## 5. Conclusions

Robotic rehabilitation induced phase-specific biomechanical adaptations that were predominantly characterized by changes in proximal trunk control and dynamic stabilization strategies. Waveform-based analysis revealed localized gait cycle adaptations that were not consistently reflected in conventional discrete parameters, highlighting the value of temporally resolved biomechanical assessment in post-stroke rehabilitation.

Exploratory PCA- and clustering-based analyses suggested inter-individual variability in biomechanical adaptation patterns, particularly among chronic stroke participants. However, given the limited sample size and overlapping clustering structure, these findings should be interpreted cautiously and considered hypothesis-generating rather than confirmatory. Overall, the findings support the utility of waveform-based biomechanical analysis for characterizing rehabilitation-induced movement reorganization following robotic gait training after stroke.

## Figures and Tables

**Table 1:** Demographic Characteristics and Stroke Phase Classification of the Study Participants.

Patient	Age (yr)	Sex	Months Since Stroke	Stroke Phase
P1	65	M	2.5	Subacute
P2	32	M	5.7	Subacute
P3	61	M	14.2	Chronic
P4	57	M	10.6	Chronic
P5	66	F	4.5	Subacute
P6	66	M	16.0	Chronic
P7	68	M	19.4	Chronic
P8	65	F	140.0	Chronic
P9	55	M	1.7	Subacute
P10	76	M	1.8	Subacute
P11	82	M	5.4	Chronic
P12	86	M	24.0	Chronic
P13	52	M	6.1	Subacute

Stroke phase classification was determined based on the time interval between stroke onset and the first rehabilitation session. Patients with stroke duration < 6 months were classified as subacute, whereas those > 6 months were classified as chronic. Borderline cases close to the 6-month threshold were classified according to clinical evaluation and rehabilitation records.

**Table 2:** Pre- and Post-Rehabilitation Comparison of Spatiotemporal and Kinematic Parameters Following 16 Sessions of Robotic Gait Training.

Parameter	Pre (μ ± σ)	Post (μ ± σ)	μ Diff	% Change	*p*-Value	Cohen d’s
Hip Flex-Ext (°)	30.06 ± 10.95	30.41 ± 7.13	+0.35	+1.2	0.901	0.04
Knee Flex-Ext (°)	39.72 ± 14.18	42.21 ± 15.97	+2.49	+6.3	0.398	0.24
Trunk Flex-Ext (°)	8.69 ± 5.03	9.69 ± 3.82	+0.99	+11.4	0.276	0.32
Trunk Lateral Flex (°)	5.08 ± 2.05	5.04 ± 1.66	−0.05	−0.9	0.943	−0.02
COG (cm)	2.97 ± 1.06	2.98 ± 1.29	+0.01	+0.4	0.945	0.02
Cadence (cycles/s)	2.61 ± 1.25	2.39 ± 0.29	−0.22	−8.4	0.982	0.47
Contact Time Left (s)	2.58 ± 1.39	2.46 ± 1.44	−0.118	−4.6	0.801	−0.07
Contact Time Right (s)	2.55 ± 1.28	2.08 ± 1.02	−0.512	−20.1	0.259	−0.34
Load Symmetry (%)	5.76 ± 5.09	5.48 ± 4.49	−0.281	−4.9	0.824	−0.058
Step Length (hemi) (cm)	17.42 ± 14.10	23.15 ± 16.05	5.73	65.8	0.024 *	0.75
Step Length (intact) (cm)	18.73 ± 15.40	27.56 ± 15.22	8.83	47.1	0.007 *	0.58

* Indicates a statistically significant difference (*p* < 0.05).

## Data Availability

Data set is available upon request.

## Abbreviations

The following abbreviations are used in this manuscript:

COG	Center of Gravity
IC	Initial Contact
LR	Loading Response
MS	Mid-Stance
TS	Terminal Stance
SW	Swing Phase
PCA	Principal Component Analysis
PC1	First Principal Component
PC2	Second Principal Component
ROM	Range of Motion
SPM	Statistical Parametric Mapping
IMU	Inertial Measurement Unit
